# A meta-analysis of the therapeutic effect of intranasal salmon calcitonin on osteoporosis

**DOI:** 10.1186/s40001-021-00610-x

**Published:** 2021-12-08

**Authors:** Ning Li, Yi Chen Gong, Jianer Chen

**Affiliations:** Zhejiang Rehabilitation Medical Center (Rehabilitation Hospital Affiliated to Zhejiang University of Traditional Chinese Medicine), Zhejiang, China

**Keywords:** Intranasal salmon calcitonin, Osteoporosis, Meta-analysis

## Abstract

**Objective:**

To evaluate the efficacy and safety of intranasal salmon calcitonin in the treatment of osteoporosis.

**Methods:**

Eight Chinese and English databases were searched by electronic search (from the establishment of the database to October 2019). The literature was screened according to the inclusion criteria and exclusion criteria, the quality was evaluated according to Cochrane software, and the Review Manager 5.2 software was used for statistical analysis.

**Results:**

A total of 374 documents were retrieved and 12 (12 original studies) were included after the screening, with a total sample capacity of 1068 cases. Meta-analysis showed that the intranasal salmon calcitonin had obvious advantages in reducing blood calcium, improving the ratio of serum creatinine and alkaline phosphatase. In addition, the intranasal salmon calcitonin had no obvious advantages in other indicators. It cannot be illustrated that the combination of intranasal salmon calcitonin and other conventional drugs is more effective than the simple use of conventional drugs.

**Conclusion:**

The intranasal salmon calcitonin is superior to conventional drugs in reducing blood calcium, increasing creatinine ratio, and alkaline phosphatase, but its advantages in other indicators such as improving the bone mineral density (BMD) of lumbar vertebrae and hip have not been confirmed, and it is not clear that the combination of intranasal salmon calcitonin and other conventional drugs is better than the simple conventional drugs.

## Introduction

With the deepening population aging process in China, primary osteoporosis (OP) has become an issue of concern to the entire society. The morbidity of osteoporosis over 60 years old increased significantly. Women, in particular, OP have increased the disability rate and mortality rate of the elderly [1]. More than half of the patients in the world are in Asia or the Pacific region.

At present, the treatment of OP is mainly dependent on drugs including oral calcium and vitamin D, but its effect of anti-osteoporosis is very weak. Other drugs such as bisphosphonates have serious adverse reactions [2], and these drugs produce a serious impact on the life and work of osteoporosis patients [3]. Since the drugs for osteoporosis need long-term treatment, the effect of short-term drug treatment is not obvious. These factors lead to low compliance of patients. For postmenopausal women with osteoporosis, hormone therapy is the most common curative treatment, but long-term use of hormones can lead to an increase in the incidence rate of myocardial infarction and breast cancer [4]. Therefore, it is very important to find a more safe and effective alternative therapy to increase BMD and prevent fracture.

The salmon calcitonin belongs to a synthetic drug composed of salmon calcitonin which can not only restrain osteoclasts in vivo, but also improve bone absorption of patients. This can not only reduce the pain of osteoporosis patients, but also reduce the incidence of adverse reactions. At present, the salmon calcitonin is mainly divided into the salmon calcitonin injection and the intranasal salmon calcitonin, and the intranasal salmon calcitonin is easier to being accepted by patients than the salmon calcitonin injection. The intranasal salmon calcitonin and conventional drugs (calcium carbonate, vitamin D) are widely used in clinics. Many patients are treated with the combination of the intranasal salmon calcitonin, calcium carbonate and vitamin D. However, whether the effect is better than a single treatment has not been confirmed. Therefore, this paper talks about the efficacy and safety analysis of the randomized controlled trials by searching for intranasal salmon calcitonin. Twelve articles about intranasal salmon calcitonin were obtained by searching each database [5–16]. This paper gives a systematic evaluation and meta-analysis of this literature to provide an objective basis for clinical medication.

## Materials and methods

### Inclusion criteria

#### Study type

Clinical research, randomized controlled trial, languages, published status and follow-up time are unlimited.

#### Research target

Includes senile osteoporosis and postmenopausal women with osteoporosis, the diagnostic standard can refer to the diagnostic standard WHO-recommended or Chinese diagnostic standard [17].

#### Interventions

To test and verify the effectiveness of the treatment of primary osteoporosis as the main purpose, the experimental group was treated with the intranasal salmon calcitonin or combine based on routine intervention in the control group; the control group may be non-intervention, placebo, or routine intervention. Routine interventions include drugs recommended by guidelines (diphosphonate, calcium, vitamin D, etc.) and other traditional Chinese medicine therapies.

#### Evaluating index

1. The primary evaluating index: fracture, quality of life, clinical symptom index (pain, etc.), adverse events/reactions [18].
2. The secondary evaluation index: BMD (lumbar and hip), blood calcium, phosphorus, and alkaline phosphatase, bone mineral content, urinary creatinine ratio, serum parathyroid hormone, blood CTX (C-telopeptide), urinary NTX (N-telopeptide).

### Exclusion criteria

Lack of research data, the research targets, interventions, and outcome indicators that did not meet the above-mentioned requirement, non-RCT literature, animal experiment, case report, repeated research, etc.

### Literature search

The English database includes PubMed, Cochrane Library, Clinical trials. Gov. The retrieval strategy is to combine the retrieval words "internal salmon calcitonin" and "osteoporosis". Chinese database includes: CNKI, WanFang, VIP, CBM, the retrieval strategy is to use the keywords of "migaixi nasal spray" and "osteoporosis" to search in different ways in every database. The maximum number of retrieved clauses and subclauses are in the outcome. The retrieval time limit of the above databases is from the establishment of the database to October 2019. The search results are imported into Noteexpress V3.0 software in the form of an inscription.

### Document extraction and quality evaluation

#### Document extraction

Two researchers extracted the retrieved literature information according to the pre-formulated criteria of literature inclusion and exclusion. The extraction content mainly includes the first author, published year, age, menopause duration, sample capacity, treatment drugs of experimental group and control group, frequency, course of treatment, evaluation criteria, adverse reactions, etc. If the information provided in the literature is not comprehensive. In case of doubt and dispute, contact the author of the document and decide careful inquiry. If there are differences, discuss and solve them. If necessary, the third researcher will provide help to solve the problem.

#### Quality evaluation

All data input system evaluation management software Review Manager 5.2. Adopting the "bias risk assessment" tool in the Cochrane assessment manual Handbook. 1.4.3. The main elements are as follows: ① generation of random sequence; ② assignment concealment; ③ blind method for study subjects and treatment plan implementers; ④ blind method in outcome evaluation; ⑤ complete outcome; ⑥ selective publication; ⑦ other bias.

### Statistical analysis

Analyze data with Review Manager 5.2 software provided by Cochrane Collaboration Network. For dichotomous variables, odds ratio (or) and 95% confidence interval (95% CI) were adopted as efficacy statistics. For continuous variables, mean difference (MD) and 95% CI were adopted as efficacy statistics, homogeneity test (q test) was adopted for heterogeneity test. When there was no statistical significance in the statistical heterogeneity among the studies (*P* > 0.10, *I*^2^ < 50%), the fixed-effect model was adopted for meta-analysis. On the contrary, when there was statistical heterogeneity among the studies (*P* < 0.10, *I*^2^ > 50%), the random effect model was considered. A funnel plot was used to evaluate and detect publication bias.

## Outcome

**Fig. 1:**
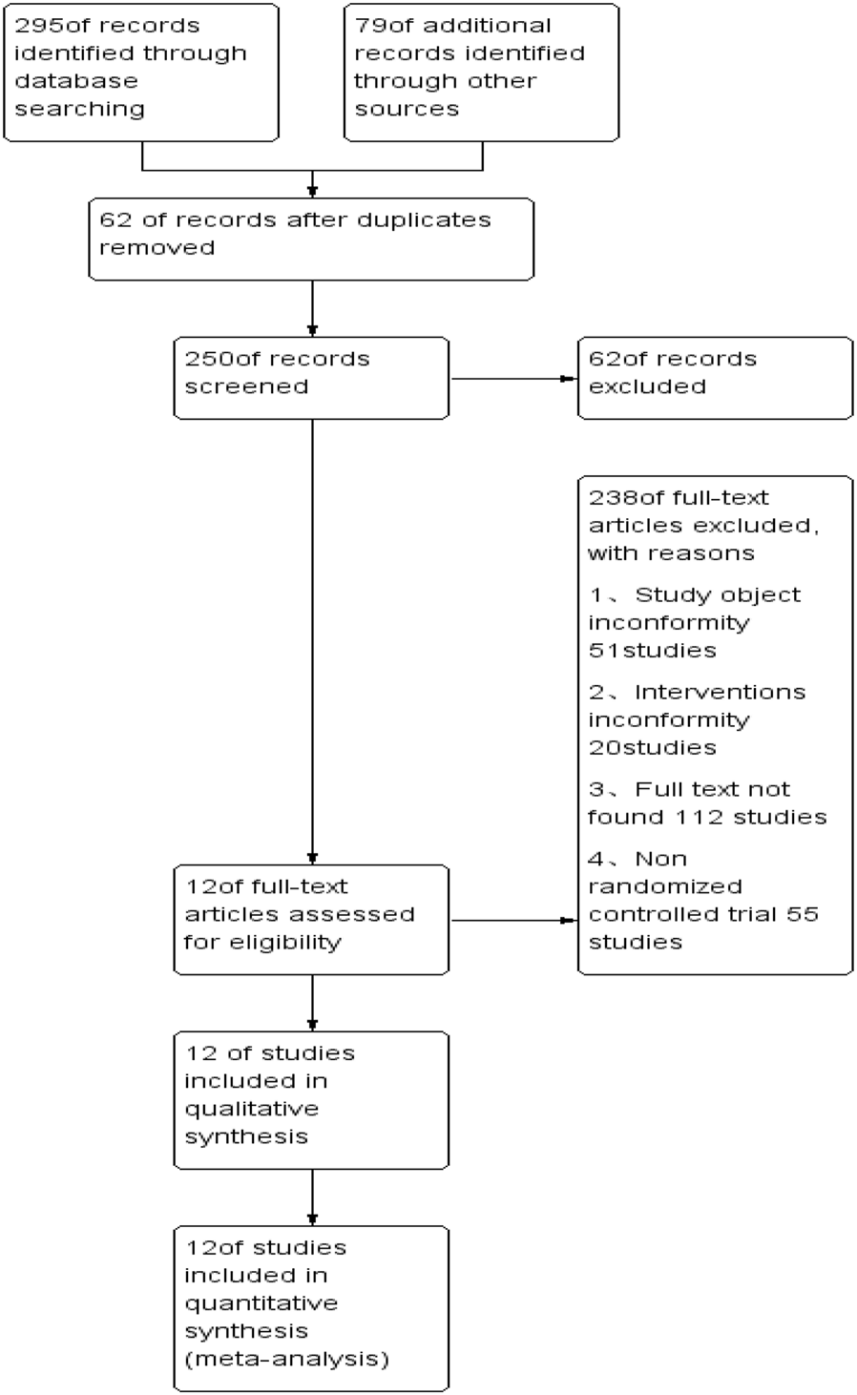
Selecting literature through databases

2.1 Document selection: A total of 374 documents were retrieved, read the abstract or the full text after checking the duplicate by Noteexpress software. The literature was screened according to the inclusion criteria and exclusion criteria. Finally, two Chinese-literature [8, 9] and ten English-literature [5–7, 10–16] were included. There are 12 randomized controlled trials reported in 12 literature, including two in China, ten abroad, and the earliest literature is published in 1989 (Fig. 1).

### Basic characteristics of the literature included

#### Research target

The 12 clinical randomized controlled trials included in this systematic evaluation included 1068 patients, all of whom were patients with primary osteoporosis. One study [9] referred to Chinese diagnostic criteria, 8 studies [6–8, 10, 11, 13–15] refer to WHO-recommended diagnostic criteria. Two studies [5, 16] are self-designed diagnostic criteria. One study [12] did not mention diagnostic criteria,12 original studies were included in this study. The minimum sample size is 35. The maximum sample size is 287. The total sample size is 1068. The experimental group is 566. The control group is 502.

#### Interventions

Among the 12 original studies, the intervention measures in the experimental group include: ① intranasal salmon calcitonin + calcium + vitamin D3:n. In one study [6], the usage and dosage was 200 IU/day (once every other month) + 500 mg calcium carbonate + 0.25 μ ɡ vitamin D3. Another study [7] was intranasal salmon calcitonin200 IU/day, calcium 1000 mg/day + 880 IU vitamin D3. In one study [11], the intranasal salmon calcitonin was given 200 IU/day + 500 mg calcium + 200 IU vitamin D3. In one study [15], the intranasal salmon calcitonin was given 100 IU/day + 1000 mg calcium + 400 IU vitamin D3; ② intranasal salmon calcitonin + calcium. In one study [8], the intranasal salmon calcitonin was given 200 IU/day + 600 mg calcium qd. In one study [12], the intranasal salmon calcitonin was given 50 IU/day five times weekly + 500 mg calcium five times weekly. In one study [13], the intranasal salmon calcitonin was given 200 IU/day + 1000 mg calcium qd. In one study [14], the intranasal salmon calcitonin was given 100 IU/day five times weekly + 500 mg calcium qd; ③ intranasal salmon calcitonin. In one study [16], the intranasal salmon calcitonin was given 400 IU/day. The intranasal salmon calcitonin was given 200 IU/day. In three studies [5, 9, 10].

#### Contrast pattern

Three studies [5, 9, 10]: intranasal salmon calcitonin vs routine interventions; onestudy [16]: intranasal salmon calcitonin vs placebo; seven studies [6, 7, 11–15]: intranasal salmon calcitonin +routine interventions vs routine interventions; onestudy [8]: intranasal salmon calcitonin +routine interventions vs salmon calcitonin injection+ routine interventions.

#### Outcome indicators: secondary outcome indicators

Five studies [6, 8, 10, 11] reported BMD of the lumbar spine. Four studies [6–8, 15] reported BMD of the hip (femoral neck, intertrochanteric, and ward triangle). Five studies [5, 6, 10, 12, 14] reported serum calcium levels. Two studies [5, 14] reported serum phosphorus level. Six studies [5–7, 10, 12, 14] reported serum alkaline phosphatase. Three studies [5–7] reported urinary creatinine ratio. Two studies [13, 16] reported bone mineral content. Two studies [6, 7] reported serum parathyroid hormone. Two studies [10, 11] reported serum CTx. Two studies [11, 14] reported urine NTx. Five studies [5, 7, 8, 12, 16] reported adverse drug reactions.

### Bias risk assessment

**Fig. 2:**
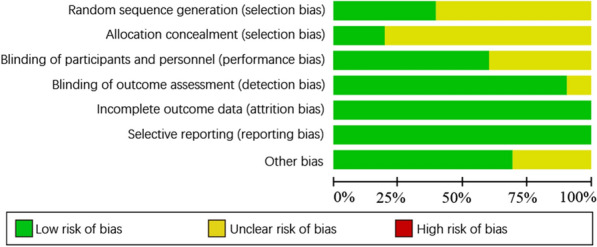
The methodological quality evaluation in all of studies included

Among 12 studies, 7 studies [5–7, 9, 13–15] showed the generation of random sequences, the rest are not illustrated. Three studies [7, 13, 14] mentioned distribution concealment and double blindness. One study [15] was distribution concealment and single-blind, and the rest are not involved. Five studies [6, 7, 13, 15, 16] reported shedding cases and causes, and all of the rest had complete outcome reports. None of the studies tells whether outcome indicators adopt the blind method or not, and it was not clear whether there was selective publication or other bias. According to the Cochrane manual "assessment tool for bias risk of randomized controlled trials (version 5.3.5)", the methodological quality evaluation is conducted in all of the studies included (Fig. 2).

### Efficacy analysis

#### Lumbar spine BMD

**Fig. 3:**
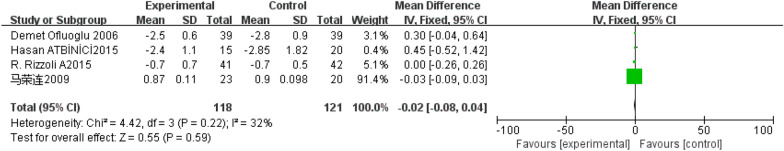
Lumbar spine BMD about 5 studies

Five studies [6, 8, 10, 11] report BMD of the lumbar spine. The results of the heterogeneity test showed that *P* = 0.01, *I*^2^ = 99%. The heterogeneity is large, and sensitivity analysis finds that intranasal salmon calcitonin is sprayed once every other month in one study and once q day in the other studies. This may be the main cause of heterogeneity. After rejecting this study, the results of the heterogeneity test showed that *P* = 0.22, *I*^2^ = 32% (Fig. 3). Therefore, using the fixed-effect model, 95% CI (- 0.08, 0.04), *P* = 0.59, there was no significant difference between the two groups, which could not explain that the treatment group of intranasal salmon calcitonin was superior to the conventional treatment group. Descriptive analysis of one study [6] was not included in the synthesis: the total sample size was 102 cases, 57 cases in the experimental group, and 45 cases in the control group. After treatment, the BMD of the experimental group and the control group were (3.0 ± 1.1) g/cm2 and (- 0.4 ± 0.6) g/cm^2^, respectively, *P* = 0.009. The difference between the two groups was statistically significant, and the treatment group was significantly better than the conventional treatment group.

#### Hip BMD

**Fig. 4:**
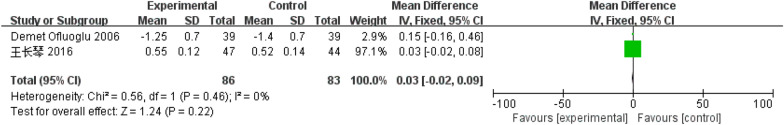
Hip BMD about 2 studies

Four studies [6–8, 15] reported hip BMD (femoral neck, intertrochanteric, and ward triangle). The results of the heterogeneity test showed that *P* = 0.00001, *I*^2^ = 100%; there is obvious heterogeneity. The reason may be related to the course of treatment. Therefore, according to the length of the course of treatment (within 6 months and more than 6 months), it is analyzed, respectively. Among the studies, the course of treatment of two [8, 15] was within 6 months (*P* =0.46, *I*^2^ =0%) (Fig. 4). Therefore, by using the fixed-effect model, 95%CI (− 0. 02,0. 09), *P* =0. 22), there was no statistical significance between the two groups. The course of treatment of the other two studies is more than 6months, P<0.00001, *I*^2^ =100%. The heterogeneity is obvious, descriptive analysis shows that the total sample size of one study [6] was 102, 57 cases in the experimental group and 45 cases in the control group. After treatment, the BMD of the hip in the experimental group and the control group were (3.1 ± 0.5) mmol/L and (− 0.8 ± 0.6) mmol/L, respectively, 95% CI (3.68, 4.12), *P* < 0.0005. The difference between the two groups was statistically significant, and the intranasal salmon calcitonin treatment group was significantly better than the conventional treatment group. The total sample size of one study [7] was 90. There were 45 cases in the experimental group and 45 cases in the control group. After treatment, the BMD of hip in the experimental group and the control group were (− 2.1 ± 0.5) mmol/L and (- 2.4 ± 0.5) mmol/L, respectively, P > 0.05. There was no significant difference between the two groups.

**Fig. 5:**
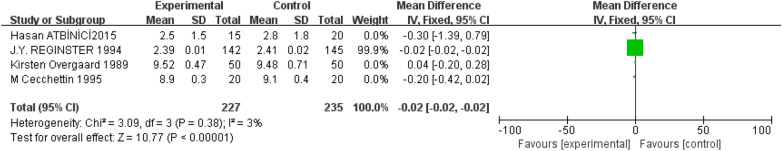
Serum calcium levels about 4 studies

2.4.3 Five studies [5, 6, 10, 12, 14] reported serum calcium levels. The results of the heterogeneity test showed that *P* < 0.00001, *I*^2^ = 93%, there was significant heterogeneity, and sensitivity analysis finds that intranasal salmon calcitonin is sprayed once every other month in one study [6]and once q day in the other studies. This may be the main cause of heterogeneity. After rejecting this study, the results of the heterogeneity test showed that *P* = 0.38, *I*^2^ = 3% (Fig. 5). Therefore, using the fixed-effect model, 95% CI (− 0.02, − 0.02), *P* <0. 0001. The difference between the two groups was statistically significant. The intranasal salmon calcitonin treatment group was significantly better than the conventional treatment group. Descriptive analysis of one study [6] was not included in the synthesis: The total sample size was 102 cases, 57 cases in the experimental group, and 45 cases in the control group. After treatment, the BMD of the experimental group and the control group were (− 0.7 ± 0.5) g/cm^2^ and (0.2 ± 0.7) g/cm^2^, respectively, *P* > 0.05. There was no significant difference between the two groups. The intranasal salmon calcitonin treatment group was not superior to the conventional treatment group.

**Fig. 6:**
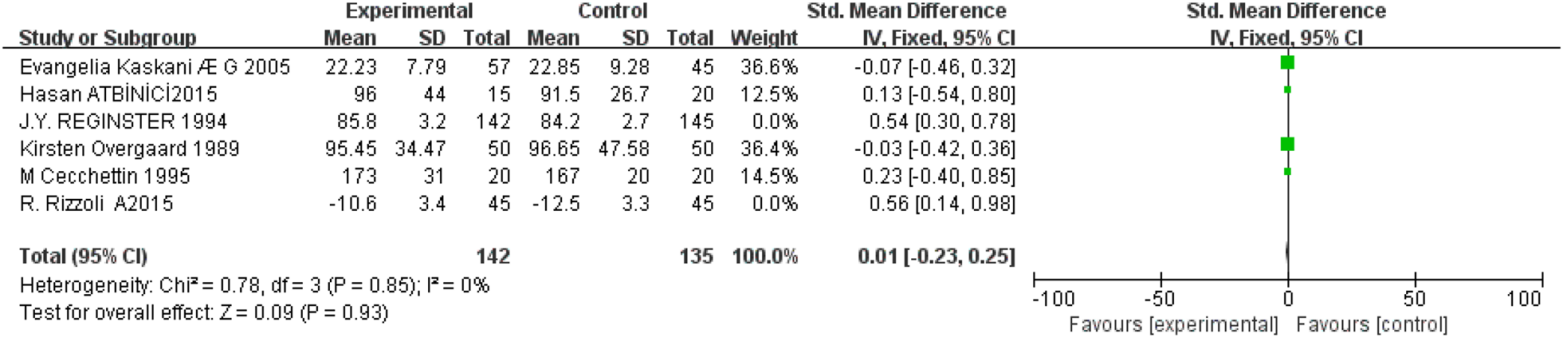
Serum alkaline phosphatase about 4 studies

**Fig. 7:**
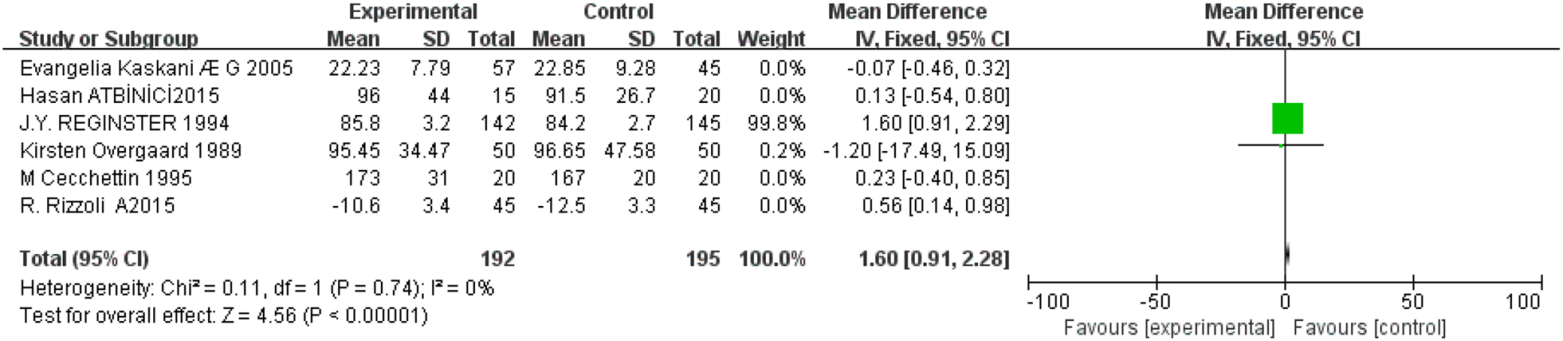
Serum alkaline phosphatase about 2 studies

Six studies [5–7, 10, 12, 14] reported serum alkaline phosphatase. The results of the heterogeneity test showed that *P* = 0.04, *I*^2^ = 58%. There was significant heterogeneity, considering that the dosage and times of the application of intranasal salmon calcitonin are the main factors affecting the results. Therefore, it is divided into group 1 [5–7, 10] and group 2 [12, 14], Group1: intranasal salmon calcitonin is sprayed once every other month. The results of the heterogeneity test showed that *P* = 0.85, *I*^2^ = 0% (Fig. 6), so fixed-effect model was used, MD = 0.78, 95% CI (- 0.23, 0.25), *P* = 0.93, there was no significant difference between the two groups. In group 2, 50 IU or 100 IU intranasal salmon calcitonin was used five times a week. The results of the heterogeneity test showed that *P* = 0.74, *I*^2^  = 0% (Fig. 7), so the fixed-effect model was used, MD =  0.11, 95% CI (0.91, 2.28), *P*  < 0.00001, the difference between the two groups was statistically significant, and the intranasal salmon calcitonin treatment group was significantly better than the conventional treatment group.

#### Serum phosphorus level

**Fig. 8.**
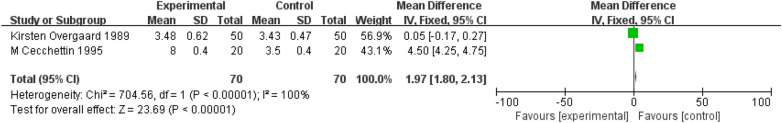
Serum phosphorus level about 2 studies

Two studies [5, 14] reported serum phosphorus levels. The results of the heterogeneity test showed that *P* < 0.00001, *I*^2^ = 100% (Fig. 8), there was significant heterogeneity. Descriptive analysis shows that one study [5] there were 40 samples in total, 20 in the experimental group and 20 in the control group. After treatment, the serum phosphorus levels in the experimental group and the control group were (8 ± 0.4) mmol/L and (3.5 ± 0.4) mmol/L, respectively, *P* > 0.05. There was no significant difference between the two groups. It cannot be concluded that the intranasal salmon calcitonin treatment group was superior to the conventional treatment group. One study [14]: 52 samples in total, 26 in the experimental group and 26 in the control group. After treatment, the serum phosphorus levels in the experimental group and the control group were (3.48 ± 0.62) mmol/L and (3.43 ± 0.47) mmol/L, respectively, *P* > 0.05. There was no significant difference between the two groups, which could not be concluded that the intranasal salmon calcitonin treatment group was superior to the conventional treatment group.

**Fig. 9:**
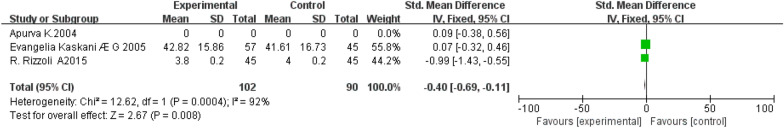
Serum parathyroid hormone level about 2 studies

2.4.6 Two studies [6, 7] reported serum parathyroid hormone levels. The results of the heterogeneity test showed that *P* = 0.0004, *I*^2^ = 92% (Fig. 9), there was significant heterogeneity. Descriptive analysis shows that one study [6]: 102 samples in total, 57 in the experimental group and 45 in the control group. After treatment, the serum phosphorus levels in the experimental group and the control group were (− 2.5 ± 2.6) pg/ml and (0.8 ± 0.4) pg/ml, respectively, *P* < 0.05. The difference between the two groups was statistically significant, and the intranasal salmon calcitonin treatment group was superior to the conventional treatment group. One study [7]: 90 samples in total, 45 in the experimental group, and 45 in the control group. After treatment, the serum phosphorus levels in the experimental group and the control group were (31 ± 11.1) pmol/L and (7.2 ± 6.2) pmol/L, respectively, *P* < 0.05. The difference between the two groups was statistically significant, and the intranasal salmon calcitonin treatment group was superior to the conventional treatment group.

**Fig. 10:**
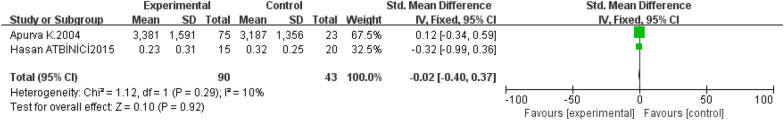
Serum CTX level about 2 studies

Two studies [10, 11] reported the serum CTX level, the heterogeneity test results showed that *P* = 0.92, *I*^2^ = 10% (Fig. 10), so the fixed-effect model was used, OR = 1.12, 95% CI (− 0.4, 0.37), *P* = 0.92, there was no statistical difference between the two groups, which could not show that the intranasal salmon calcitonin treatment group was superior to the conventional treatment group.

**Fig. 11:**
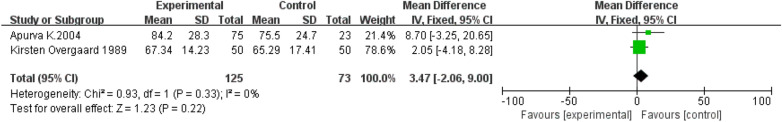
Urine NTx level about 2 studies

Two studies [11, 14] reported the urine NTx level, the heterogeneity test results showed that *P* = 0.33, *I*^2^ = 0% (Fig. 11), so the fixed-effect model was used, 95% CI (− 2.06, 9.00), *P* = 0.22, there was no statistical difference between the two groups, which could not show that the intranasal salmon calcitonin treatment group was superior to the conventional treatment group.

**Fig. 12:**
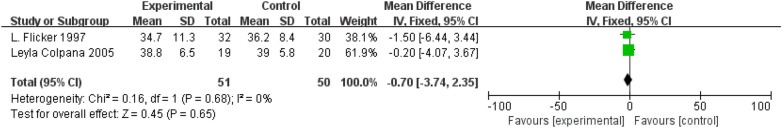
Bone mineral content level about 2 studies

Two studies [13, 16] reported the bone mineral content level, the heterogeneity test results showed that *P* = 0.68, *I*^2^  = 0% (Fig. 12), so the fixed-effect model was used, 95% CI (− 3.74, 2.35), *P* = 0.65, there was no statistical difference between the two groups, which could not show that the intranasal salmon calcitonin treatment group was superior to the conventional treatment group.

**Fig. 13:**
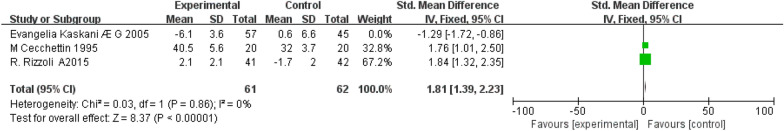
Urinary creatinine ratio level about 2 studies

3 studies [5–7] report urinary creatinine ratio level. The results of the heterogeneity test showed that *P* = 0.07, *I*^2^ = 98%. The heterogeneity is large, and sensitivity analysis finds that intranasal salmon calcitonin is sprayed once every other month in one study [6] and once q day in the other studies. This may be the main cause of heterogeneity. After rejecting this study, the results of the heterogeneity test showed that *P* = 0.86, *I*^2^ = 0% (Fig. 13). Therefore, using the fixed-effect model, 95% CI (1.39, 2.23), *P* < 0.00001, the difference between the two groups was statistically significant, and the intranasal salmon calcitonin treatment group was significantly better than the conventional treatment group. Descriptive analysis of one study [6] was not included in the synthesis: The total sample size was 102 cases, 57 cases in the experimental group, and 45 cases in the control group. After treatment, the urinary creatinine ratio of the experimental group and the control group were (− 6.1 ± 3.6) and (0.6 ± 6.6), respectively, *P* > 0.05. There was no statistical difference between the two groups, which could not show that the intranasal salmon calcitonin treatment group was superior to the conventional treatment group.

### Adverse reaction

Five studies [5, 7, 8, 12, 15] reported adverse drug reactions. Two [5, 7] of the studies report adverse drug reactions in both the study group and the control group. One study [5]: the intranasal salmon calcitonin treatment group reported one case of pruritus and one case of epistaxis, and the control group reported four cases of a stomachache. One study [7]: the intranasal salmon calcitonin treatment group reported 14 cases of arthralgia and 8 patients detected antibodies, and the control group reported 26 cases of arthralgia symptoms. One study [8]: the intranasal salmon calcitonin treatment group reported one case of nose injury and one case of skin pruritus. One study [12] reported the patients not tolerating intranasal salmon calcitonin and calcium in varying degrees in the experimental group and the control group. A case of nasal hypersensitivity was reported in the control group of one study [15].

### Publication bias assessment

Among the outcome indicators, the number of studies on each indicator is less than 7, so there is no publication bias assessment.

## Discussion

Migaixi nasal spray is a commonly used anti-osteoporosis drug in the clinic. It is widely used clinically because of its convenient carrying and non-invasive advantages. However, there are different opinions on its clinical efficacy at home and abroad. In this paper, a systematic meta-analysis of its clinical efficacy is carried out based on the research of 12 domestic and foreign literature. No main outcome indicators of primary osteoporosis were reported in the studies included. The number of fractures, types of fractures, and the number of deaths directly or indirectly caused by osteoporosis. This may be due to the biased choice of subjects and the short follow-up time. The results of efficacy evaluation show that in terms of improving lumbar BMD, there was no significant advantage in improving the BMD of lumbar vertebrae by using only intranasal salmon calcitonin (once a day) or intranasal salmon calcitonin (once a day) + routine intervention. Because of the lack of literature, the specific efficacy needs to keep on being explored, so at present, it cannot be concluded that the intranasal salmon calcitonin has obvious advantages in improving lumbar BMD. In terms of improving the BMD of the hip, within 6 months of treatment, there is no obvious advantage in the treatment of intranasal salmon calcitonin (once a day) + conventional intervention compared with conventional treatment. When the total course of treatment is more than 6 months, because the selected literature has great heterogeneity, there is no comparative significance. In conclusion, intranasal salmon calcitonin has no obvious advantage in improving the lumbar BMD and hip BMD. In the analysis of hip BMD for more than 6 months, there is a big heterogeneity problem, which may be caused by the diversity between measurement methods, measurement methods and selected objects. The analysis of serum calcium showed that the effect of only using intranasal salmon calcitonin (once a day) was better than that of routine intervention, and intranasal salmon calcitonin (once a day) + routine intervention was also better than that of routine intervention, but the clinical effect of intranasal salmon calcitonin (once a day every other month) was not clear and definite because of the sample capacity. The analysis of serum alkaline phosphatase showed that there was no significant difference in the efficacy of intranasal salmon calcitonin (once a day for 200IU) whether it was used alone or combined with routine intervention. The efficacy of intranasal salmon calcitonin (50IU or 100IU five times weekly) + routine intervention was significantly better than that of the simple routine intervention group. We can see the effect of intranasal salmon calcitonin on alkaline phosphatase is closely related to the dosage and times of intranasal salmon calcitonin application, and its specific effects need to be further discussed and analyzed. For the influence of blood phosphorus and serum parathyroid hormone level, although the two kinds of literature show that the therapeutic effect is obvious, because of the great heterogeneity of the two kinds of literature, it is not clear to illustrate the definite therapeutic effect of the intranasal salmon calcitonin. For the analysis of serum CTX, urinary NTX, and bone mineral content, it cannot be concluded that the intranasal salmon calcitonin treatment group is better than the conventional treatment group. For the ratio of urine creatinine, the intranasal salmon calcitonin treatment group was significantly better than the conventional treatment group. In conclusion, compared with the conventional drugs, the intranasal salmon calcitonin can significantly improve the blood calcium level, and the effect on the urinary creatinine ratio is also better than that of the conventional treatment group. The intranasal salmon calcitonin (50IU or 100IU five times weekly) has a better effect on alkaline phosphatase than that of the conventional treatment group. The intranasal salmon calcitonin has no obvious advantage on BMD compared with the conventional treatment drugs.

In terms of safety, there are a lot of adverse reactions to intranasal salmon calcitonin. Whether it is a single application of intranasal salmon calcitonin or a combination of intranasal salmon calcitonin and routine intervention, there are mainly pruritus, epistaxis, and arthralgia; the reactions are relatively mild, without any sequelae. This study is a simple randomized group. There is no significant analysis on the factors that may affect the effect, such as the course of treatment and the time of menopause, and there should be further exploration of large sample and high-quality research in the future.

In this study, we searched for the published literature about intranasal salmon calcitonin. However, most of the original studies are of low quality and have methodological and clinical heterogeneity. The data of minority studies have suspected mistakes. We fail to contact the original researchers to correct the suspicious data.

## Conclusion

It is suggested that in addition to blood calcium, urinary creatinine ratio, and alkaline phosphatase, there is no obvious advantage in other indicators comparing with the conventional treatment group, and whether the combination of the two treatment plans is better than the conventional drugs alone has not been confirmed, which needs further large sample study. Intranasal salmon calcitonin and other conventional drugs have different adverse reactions which should be adjusted in time according to the individual constitution of patients. This study does not recommend the combination of intranasal salmon calcitonin and conventional drugs to avoid the incidence of adverse reactions.

## Data Availability

All of the data and information about this article can be applied.

## Abbreviations

OP: Osteoporosis
Blood CTX: Blood C-telopeptide
Urinary NTX: Urinary N-telopeptide
BMD: Bone mineral densityo
